# Cost-Effectiveness of the Strategies to Reduce the Incidence of Dengue in Colima, México

**DOI:** 10.3390/ijerph14080890

**Published:** 2017-08-08

**Authors:** Oliver Mendoza-Cano, Carlos Moisés Hernandez-Suarez, Xochitl Trujillo, Héctor Ochoa Diaz-Lopez, Agustin Lugo-Radillo, Francisco Espinoza-Gomez, Miriam de la Cruz-Ruiz, Ramón Alberto Sánchez-Piña, Efrén Murillo-Zamora

**Affiliations:** 1 Facultad de Ingeniería Civil, Universidad de Colima, Colima 28400, Mexico; 2 Center for Health and the Global Environment, Department of Environmental Health, Harvard TH Chan School of Public Health, Boston, MA 02215, USA; rsanchez@hsph.harvard.edu; 3 Facultad de Ciencias, Universidad de Colima, Colima 28045, Mexico; carlosmh@mac.com; 4 Centro Universitario de Investigaciones Biomédicas, Universidad de Colima, Colima 28045, Mexico; rosio@ucol.mx; 5 El Colegio de la Frontera Sur Unidad San Cristóbal, Carretera Panamericana y Periférico Sur s/n, Barrio María Auxiliadora, San Cristóbal de Las Casas, Chiapas 29290, Mexico; hochoa@ecosur.mx; 6 CONACYT-Facultad de Cirugía y Medicina, Universidad Autónoma Benito Juárez de Oaxaca, Oaxaca 68020, Mexico; alugora@conacyt.mx; 7 Facultad de Medicina, Universidad de Colima, Colima 28045, Mexico; fespin@ucol.mx (F.E.-G.); ffrescky@hotmail.com (M.d.l.C.-R.); 8 Coordinación de Vigilancia Epidemiológica, Jefatura de Prestaciones Médicas, Instituto Mexicano del Seguro Social, Colima 28000, Mexico; efren.murilloza@imss.gob.mx

**Keywords:** dengue, economic analysis, ULV spraying, community participation

## Abstract

Dengue fever is considered to be one of the most important arboviral diseases globally. Unsuccessful vector-control strategies might be due to the lack of sustainable community participation. The state of Colima, located in the Western region of Mexico, is a dengue-endemic area despite vector-control activities implemented, which may be due to an insufficient health economic analysis of these interventions. A randomized controlled community trial took place in five urban municipalities where 24 clusters were included. The study groups (*n* = 4) included an intervention to improve the community participation in vector control (A), ultra-low volume (ULV) spraying (B), both interventions (AB), and a control group. The main outcomes investigated were dengue cumulative incidence, disability-adjusted life years (DALYs), and the direct costs per intervention. The cumulative incidence of dengue was 17.4%, A; 14.3%, B; 14.4%, AB; and 30.2% in the control group. The highest efficiency and effectiveness were observed in group B (0.526 and 6.97, respectively) and intervention A was more likely to be cost-effective ($3952.84 per DALY avoided) followed by intervention B ($4472.09 per DALY avoided). Our findings suggest that efforts to improve community participation in vector control and ULV-spraying alone are cost-effective and may be useful to reduce the vector density and dengue incidence.

## 1. Introduction

Dengue is an epidemic disease transmitted to humans by the bite of infected *Aedes (Ae.) aegypti* and *Ae. albopictus* mosquitoes [1]. The disease represents a growing public health concern worldwide and is the most important vector-borne viral illness due to its high incidence rates [2]. Approximately two-thirds of the world population reside in the areas prone to dengue transmission [3].

Since dengue introduction to Mexico in the late seventies, the highest incidence rates have been observed among working age individuals [4]. Currently, there is not an approved dengue vaccine and the prevention of this viral disease is focused on the reduction of bites by infected mosquitoes [5]. Dengue control measures include ultra-low (ULV) spraying with insecticides, environmental sanitation (cleaning campaigns to retire rainwater containers/trash), and consciousness raising (media programs); the implementation costs are high and a limited benefit has been documented [6].

The economic assessment (e.g., cost-effectiveness analysis using Disability-Adjusted Life Year, DALY) of interventions focusing on dengue prevention is helpful to evaluate the effectiveness of interventions. To our best knowledge, there are no published studies evaluating the effectiveness of dengue control strategies and the economic burden of the disease in endemic areas from Mexico.

The aim of this study is to evaluate, from a cost-effectiveness perspective, three different strategies: community participation, ULV spraying, and the combination of both.

## 2. Methods

**Figure 1:**
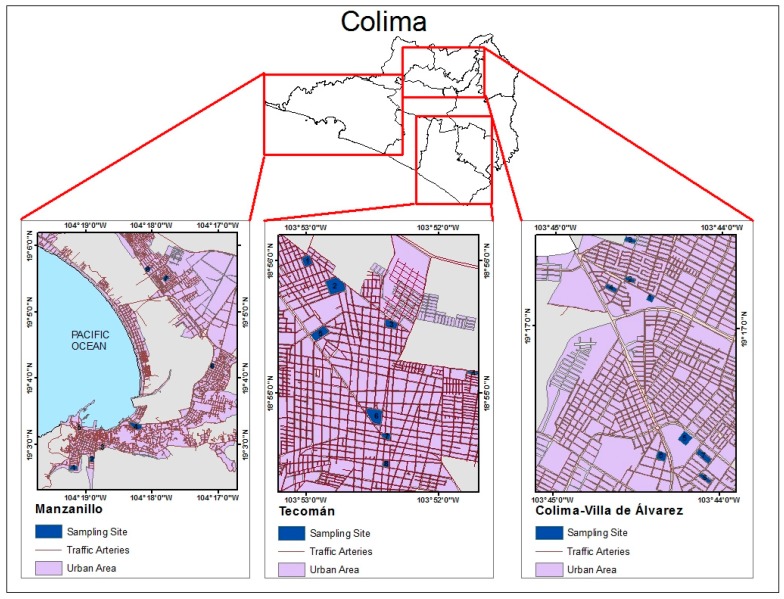
Randomized clusters of the study in the state of Colima, Mexico. Shaded blocks (sampling sites) in three study groups (Colima-Villa de Álvarez, Tecomán, and Manzanillo). The mean number of inhabitants per block was 3000.

**Figure 2:**
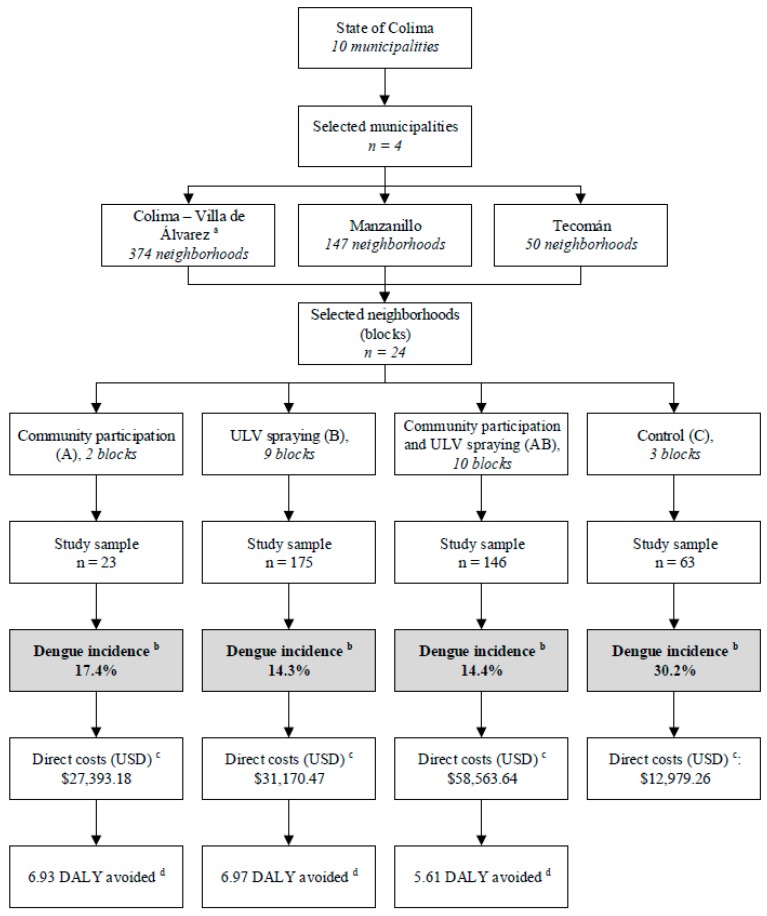
Study profile. Abbreviations: ULV, ultra-low volume; USD, United States dollar; DALY: disability-adjusted life years. ^a^ Both municipalities were clustered due to their geographical nearness; ^b^ The cumulative incidence of laboratory-confirmed dengue cases is presented; ^c^ The total implementation costs are shown; ^d^ Dengue-associated DALY per intervention group.

A randomized controlled community trial took place from February to August 2008, considering a population of 592,000 habitants and an estimated incidence of 5% in five months for this region [7], and a minimum reduction of 25% with the intervention in four urban municipalities in Colima state: Colima, Villa de Álvarez, Tecomán, and Manzanillo. The selected municipalities were those with the highest population density in the state. A randomized multistage cluster sampling was used to select the study sampling (Figure 1). First, the municipalities were grouped in three (Colima-Villa de Álvarez, Tecomán, and Manzanillo) according to their geographical location in the most populated cities in the state. Colima-Villa de Álvarez were grouped together because they are co-urbanized. Second, eight clusters or neighborhoods (mean area 5000 m^2^ each) were selected using a simple random procedure; the clusters were classified as residential or non-residential (commercial zones) blocks. Third, 10 houses were randomly selected by a simple random procedure from each block (sampling site), and their inhabitants, regardless of sex or age group, self-referring diurnal stay at home for at least 8 h were potentially eligible to participate in the study. Finally, the selected blocks and their residing individuals were allocated into the study groups. In order to perform the basal and follow-up serological testing, 40 peripheral blood samples were collected from each participating block. The study groups (*n* = 4) included a strategy of community participation (A), ultra-low volume (ULV) spraying (B), both interventions (AB), and a control group. The participating blocks were randomly allocated to the study groups (Figure 2).

The strategy that promotes community participation (A) in dengue control included the diffusion of printed materials regarding the prevention of vector proliferation, the integration of local discussion groups involving community leaders, and group visits performed in randomly selected homes to identify and show homeowners potential *Ae*. breeding sites. Encounters with local leaders and other stakeholders were made about five times in each block, meanwhile local visits conducted twice during the study. The participants represented about 15% (10 to 20%) of the total habitants per block. In addition, a play called “Looking for Pepe the Mosquito” was performed to promote the involvement of all community members in preventive actions; this play was presented to 60 people in each block. An evaluation was performed and validated for all community participation strategies [8]. The Ministry of Health (MOH) in Colima broadcasted media to alert the public to take immediate action to empty, eliminate, or clean all containers that hold water. This massive promotion included the program called “Patio Limpio”, performed by the MOH over several years, although its range of specific coverage has never been estimated.

According to the Federal Mexican Normative standards, the ULV spraying (B) was performed in Mexico using permethrin and piperonyl butoxide with 11.1 g of active ingredient per hectare, respectively (drops sized 40–50 microns) [9]. We recorded the application of spatial fumigations that were performed by the MOH during at least one week of the study period. The houses and individuals considered as treated with (B) were those who reported the spraying in front of their houses or registered on the routes reported by the MOH. Blocks where neither ULV spraying nor informative campaigns took place during the study period represented the control group. The follow-up for the control group was the same and simultaneous with that for the treated groups.

The rates of incidence were calculated by dividing the number of new cases (positive to IgM) by the total sample by groups, following which the Rates Ratio with its respective confidence intervals at 95% was analyzed by the Rates Ratio estimated by means of a bivariate logistic regression. Dengue cumulative incidence and DALYs avoided per study group (A, B, AB, and control) were the main outcomes investigated. At the end of the study, the resultant clusters and interventions were as follows: (Colima-Villa de Alvarez: 2B, 3AB, 1A, and 2C; Tecomán: 4B, 4AB; Manzanillo: 3B, 3AB, 1A, and 1C).The direct costs associated with each intervention were also computed. We conducted the Consolidated Health Economic Evaluation Reporting Standards (CHEERS) Task Force Report for this research.

This study was approved by the Research Ethics Committee from the MOH (142/09). The participants and parents or legal guardians of child/minor participants provided written, informed consent to participate in the study.

### 2.1. Direct Costs

The direct operative costs of implementing each intervention were collected according to each strategy undertaken during the fieldwork. The group undergoing intervention A was composed of five people, for which salaries, materials, and field expenses were calculated. Group B, which underwent ULV spraying as an intervention strategy, was calculated based on cost assumptions for the MOH to implement ULV spraying according to Federal Mexican Normative standards [8]. The cost for group AB were the sum of those strategies, while only surveillance costs were included for the control group. A total of eight months (preparation, one month; implementation, six months; data analysis, one month) were considered in cost computing. We employed United States Dollars in this study, equivalent to Mexican Pesos at an Exchange Annual Sale for 2008. Table 1 shows the costs of each intervention in this study.

### 2.2. Efficacy

Dengue rates were used to evaluate the efficacy of each intervention using the number of laboratory-confirmed incident cases after the follow-up (seven months). Individuals with negative circulating antibodies at the baseline measurement and positive serology testing after the follow-up were considered as having been infected during the study period. Determination of circulating antibodies against dengue virus was performed using immunochromatographic rapid tests (Panbio^®^ Dengue Duo Cassette, Panbio Limited, Queensland, AU, Spain). Dried capillary blood samples on filter paper were used. The risks ratios and efficiency parameters in each group were calculated.

### 2.3. Disability-Adjusted Life Years (DALYs)

DALYs were calculated based on the 2008 projections from the National Population Council (CONAPO, acronym in Spanish). The total population of the state, by sex and age group, was obtained from the National Institute of Geography and Statistics (INEGI, acronym in Spanish). The life expectancy for males and females aged 0–19 and 20–79 years was 75.3 and 75.5 years, respectively. The disease durations and specific disability weights for countries categorized as Established Market Economics were used. The next parameters were fixed: discount rate (*r*) = 0.03, age-weighting (*β*) = 0.04, adjustment constant for age-weights (*C*) = 0.1658, and age-weighting modulation (*K*) = 0. The number of confirmed Dengue Fever (DF, *n* = 4122) and Dengue Hemorrhagic Fever (DHF, *n* = 356) cases during 2008 in Colima state were used to compute the age-adjusted rates and the likelihood to develop DHF (0.0794).

We considered 3000 inhabitants as a population size for each strategy regarding the costs of DF and DHF. The number of infected and hospitalized cases in 2008 in Colima, as well as the mortality and years lost per person were calculated to obtain years of life lost (YLL) and years lost due to disability (YLD). The age-adjusted rates were evaluated from 0–19 years, 20–39 years, 40–59 years, and 60–79 years or more. We also considered the medical costs from the Institutional Expenses Field of hospitalization from each of the social security Institutions in Colima (Instituto Mexicano del Seguro Social (IMSS), Instituto de Seguridad y Servicios Sociales de los Trabajadores del Estado (ISSSTE), and Ministry of Health (MOH)).

For DHF we considered emergency care, medications, blood, and blood products as well as an average of three days in intensive care, two in inpatient care, and four days of hospitalization, depending on the social security institutions attended.

A cost-effectiveness approach (directs costs per DALYs avoided) was used to evaluate the implemented interventions.

## 3. Results

The total direct costs derived from each group (A, B, AB, and C) are shown in Table 1. A two-fold increase in the implementation costs was observed in group A, where the lowest cost was observed ($27,393.18) when compared with the control group ($12,979.29).

The number of laboratory-confirmed incident cases of dengue infection was 4, 25, 21, and 19 in groups A, B, AB, and C, respectively. Table 2 shows the cumulative incidence of dengue per group. The incidence of the vector-borne disease was similar between groups B and AB (14.3% and 14.4%), but the direct costs from group AB were considerably higher ($58,563.64 vs. $31,110.47). The highest efficiency and effectiveness estimates were observed in group B (0.526 and 6.97, respectively). However, the cost-effectiveness balance shown in Table 3 reveals that the strategy of community participation (A) was more cost-effective ($3952.84 per DALY avoided).

## 4. Discussion

The cost-effectiveness of different strategies of dengue control programs was evaluated in an endemic area. We found that the strategy designed to improve community participation in vector control resulted in better cost-effectiveness.

The autochthonous transmission of chikungunya and zika virus also transmitted to humans by the bite of *Ae*. Mosquitos, have been evidenced in Mexico [10,11]. These emergent diseases highlight the need for effective vector-control interventions.

Published data suggests that community-based strategies have the highest effectiveness in mosquito’s control [12]. However, the current scientific data regarding the relationship between dengue knowledge and household prevention of the vector proliferation is controversial. Some studies have documented a positive relationship [13,14] and others have not observed any correlation between disease knowledge and *Ae*. levels [15]. These findings may have been determined by discrepancies in the operationalization of knowledge.

Educational interventions were described combined with Malathion fumigation [16], and are associated with a decreased efficacy, perhaps due the false expectations regarding the benefits of spraying. This may potentially minimize the community effort to eliminate breeding spots of *Ae. aegypti*. This could be an explanation of the behavior observed in the present study, where the combined AB strategy exhibited lower results than A or B alone. Other experiences in Asia have also shown that integrated vector-control strategy based on community involvement was effective in the prevention and control of dengue fever epidemics [17]. As shown in Table 3, the 3952.84 value of A and 4472.09 value of B $ per DALY present a cost-effective balance that is comparable [18] with environmental vector control (more than $2000 USD per DALY averted). Also shown in Table 3, the calculated values and the observed differences between treatments actually mean that community participation is likely to present better cost-effectiveness in terms of operational dengue and mosquito control (3952.84 of A and 4472.09 of B $ per DALY). The MOH should take this into consideration and consider community participation as a strategy that would work for public health.

Further to the fact that the cost-effective balance shows that community participation is likely to be more cost-effective compared to other strategies, we must note that sustainability is also a challenge in disease control programs. The community participation strategy is more sustainable as it does not use chemicals compounds. In Santiago de Cuba between 2001 and 2002, a community-based dengue control intervention was more effective than spraying, providing additional evidence that these educational interventions seem to be sustainable [19]. The long-term use of chemical compounds may affect their own effectiveness, because arthropods are able to modify their metabolic mechanism leading to insecticide resistance [20,21]. Moreover, the presence of other compounds (i.e., agrochemicals) and urban or industrial pollutants in mosquito breeding sites increase tolerance to pyrethroids [22].

Community health can be also affected by pesticides exposure. The exposure to pesticides in rural environments may lead to impaired immune function [23]. As observed in animal models, pyrethroids cross the placental barrier and may interfere with fetal development; in fact, high levels of these compounds have been found among pregnant women [24]. Studies that deploy genetic strategies against diseases such as malaria, dengue, zika, or chikungunya face a more immediate hurdle: such methods may not work in the near future. As insects evolve their resistance to new pesticides, so gene traits evolve too; this strategy may therefore produce resistance and may do so far faster than suspected [25]. So, these new genetic techniques to control *Ae. aegypti* may not be ready for use on a large scale until proven safe for public health.

## 5. Conclusions

The presence of *Ae. aegypti* is continuous in tropical and subtropical areas of Mexico, and vector-borne diseases are among the major causes of morbidity in these populations. Our findings suggest that the implementation of strategies to promote community participation, as well as ULV-spraying alone are effective in vector control. It must be remarked that this study analyzed the effect of interventions directly upon dengue incidence in people, independent of *A aegypti* density. The community-based intervention was more likely to offer the advantage of better cost-effectiveness, and the potential risks of human exposure to chemical compounds used in nebulization are be minimized. Sustainability must also be considered by decisionmakers when they implement a specific vector-borne disease control program, since it carries environmental, economic, and social implications. More than ever, community participation on vector-borne diseases must be given public health importance. This is evidenced by the strategy’s cost-effectiveness and sustainability, and given that, this strategy avoids inciting potential resistance to new pesticides and will not be altered by the lack of success of genetic modifications.

## Figures and Tables

**Table 1:** Directs costs per vector-control intervention.

Item	Intervention Phases				Cost ($)	
	P/C	I	E	Co.	Unit	Total
**Community participation (A)**						
Instructors (hours)	170	480	200	240	4.94	5381.51
Students (hours)	-	480	100	240	1.25	1030.52
Community leaders (hours)	-	240	-	-	3.59	3447.04
Support staff (months)	0.5	2	1	-	1077.20	3770.20
Printed materials (units)	66	360	140	-	0.45	254.04
Computers rent (hours)	100	160	50	40	0.90	314.18
Headquarters rent (months)	2	2	2	2	538.60	4308.80
Food/Transportation allowance (day)	25	40	10	35	26.93	8886.89
*Total*						27,393.18
**ULV spraying (B)**						
Coordinators (hours)	20	200	50	-	4.94	7271.10
Technical staff (hours)	40	320	-	-	1.25	705.48
Community leaders (hours)	-	440	-	-	0.90	394.97
ULV equipment and pesticide (hours)	-	480	-	-	26.93	12,926.39
Pick-up vehicle (day)	-	15	-	-	134.65	2356.37
Headquarter rent (month)	2	4	2	-	538.60	5026.93
Food/Transportation allowance (day)	10	15	10	-	35.91	2513.46
*Total*						31,170.47
**Community participation and ULV spraying (AB)**						
*Total*						58,563.64
**Control (C)**						
Coordinators (hours)	20	200	50	-	4.94	7271.10
Technical staff (hours)	40	320	-	-	1.25	681.24
Headquarter rent (month)	2	4	2	-	538.60	5026.93
*Total*						12,979.26

Abbreviations: P/C, planning/capacitation; I, implementation; E, evaluation; Co., communication; ULV, ultra-low volume.

**Table 2:** Efficiency and effectiveness of vector-control interventions.

Group	Cases Tested/Positives	Incidence ^a^	Incidence Treated by C ^b^	Efficiency ^c^	Effectiveness ^d^
A	23/4	17.4% (12.6–24)	0.58	0.423	6.93
B	175/25	14.3% (9.3–19.3)	0.47	0.526	6.97
AB	146/20	14.4% (9.4–19.2)	0.48	0.523	5.61
C	63/19	30.2% (20–40)	1.00	0	0

Abbreviations: A, community participation; B, ultra-low volume (ULV) nebulization, AB, community participation and ULV fumigation; C, control; ^a^ The cumulative incidence; ^b^ Incidence treated by C = Incidence Ratio Treatment/Control; ^c^ Efficiency = 1-Incidence treated by C; ^d^ Avoided DALY (Disability-Adjusted Life Year).

**Table 3:** Cost-effectiveness balance.

Group	Costs ($) per DALY ^a^
A	3952.84
B	4472.09
AB	10,439.15
C	-

Abbreviations: DALY, Disability-Adjusted Life Year; ^a^ The direct costs associated with the interventions per DALY avoided are presented.
